# Mild hypothermia pretreatment improves hepatic ischemia-reperfusion injury: A systematic review and meta-analysis of animal experiments

**DOI:** 10.1371/journal.pone.0305213

**Published:** 2024-07-02

**Authors:** Li-juan Wei, Ke Wei, Shu-yu Lu, Min Wang, Chun-xi Chen, Hui-qiao Huang, Xiao Pan, Pin-yue Tao

**Affiliations:** 1 Department of Anaesthesiology, The Second Affiliated Hospital of Guangxi Medical University, Nanning, Guangxi, China; 2 Department of Nursing, The Second Affiliated Hospital of Guangxi Medical University, Nanning, Guangxi, China; 3 Department of Otorhinolaryngology, The Second Affiliated Hospital of Guangxi Medical University, Nanning, Guangxi, China; University of Cambridge, UNITED KINGDOM

## Abstract

**Background and aim:**

Mild hypothermia in hepatic ischemia-reperfusion injury is increasingly being studied. This study aimed to conduct a systematic evaluation of the effectiveness of mild hypothermia in improving hepatic ischemia-reperfusion injury.

**Methods:**

We systematically searched CNKI, WanFang Data, PubMed, Embase, and Web of Science for original studies that used animal experiments to determine how mild hypothermia(32–34°C) pretreatment improves hepatic ischemia-reperfusion injury(in situ 70% liver IR model). The search period ranged from the inception of the databases to May 5, 2023. Two researchers independently filtered the literature, extracted the data, and assessed the risk of bias incorporated into the study. The meta-analysis was performed using RevMan 5.4.1 and Stata 15 software.

**Results:**

Eight randomized controlled trials (RCTs) involving a total of 117 rats/mice were included. The results showed that the ALT levels in the mild hypothermia pretreatment group were significantly lower than those in the normothermic control group [Standardized Mean Difference (SMD) = -5.94, 95% CI(-8.09, -3.78), *P*<0.001], and AST levels in the mild hypothermia pretreatment group were significantly lower than those in the normothermic control group [SMD = -4.45, 95% CI (-6.10, -2.78), *P*<0.001]. The hepatocyte apoptosis rate in the mild hypothermia pretreatment group was significantly lower than that in the normothermic control group [SMD = -6.86, 95% CI (-10.38, -3.33), *P*<0.001]. Hepatocyte pathology score in the mild hypothermia pretreatment group was significantly lower than that in the normothermic control group [SMD = -4.36, 95% CI (-5.78, -2.95), *P*<0.001]. There was no significant difference in MPO levels between the mild hypothermia preconditioning group and the normothermic control group [SMD = -4.83, 95% CI (-11.26, 1.60), *P* = 0.14]. SOD levels in the mild hypothermia preconditioning group were significantly higher than those in the normothermic control group [SMD = 3.21, 95% CI (1.27, 5.14), *P* = 0.001]. MDA levels in the mild hypothermia pretreatment group were significantly lower than those in the normothermic control group [SMD = -4.06, 95% CI (-7.06, -1.07) *P* = 0.008].

**Conclusion:**

Mild hypothermia can attenuate hepatic ischemia-reperfusion injury, effectively reduce oxidative stress and inflammatory response, prevent hepatocyte apoptosis, and protect liver function.

## 1. Introduction

Hepatic ischemia-reperfusion injury (HIRI) is characterized by a phenomenon in which the damage to liver cells, including ischemia, worsens and is not alleviated after the restoration of blood supply. It is primarily driven by hepatocyte hypoxia, resulting in mitochondrial dysfunction, reduced ATP production, and the generation of reactive oxygen species, thereby leading to oxidative stress, endothelial dysfunction, DNA damage, and inflammatory cascades and ultimately causing autophagy, apoptosis, and necrosis in hepatocytes [1]. Liver transplantation is the most effective treatment for end-stage liver disease, but HIRI is an unavoidable pathological injury during transplantation, which may lead to acute and chronic tissue rejection and organ damage, and is a cause of liver graft dysfunction after transplantation [2, 3]. Some studies have shown that over 70% of surgical and perioperative liver transplantation complications are associated with HIRI [4, 5]. It can be seen that HIRI is one of the drivers of delayed recovery and loss of function after liver transplantation, and currently, there are no clinically feasible agents available to protect the liver from IRI [6, 7].

Hypothermia at 4°C is widely used in organ transplantation to protect donor organs from IRI. However, exposing organs to such low temperatures in vivo can lead to serious side effects such as hypotension, arrhythmia, and metabolic acidosis [8, 9]. And mild hypothermia (32–34°C) can effectively reduce the side effects associated with deep hypothermia while providing superior protection. The widespread and well-established use of mild hypothermia treatment as a clinical physical therapy method highlights its promising application prospects. It has become an important approach in the treatment of neurological diseases and has shown good efficacy in brain protection and the alleviation of nervous system diseases [10–12]. Recently, scholars have increasingly recognized the potential of mild hypothermia to mitigate ischemia-reperfusion injury in various contexts, such as heart and nerve diseases, and its efficacy in improving ischemia-reperfusion injury in liver and renal transplant surgeries has also been demonstrated to some extent [13, 14]. This study enhances our understanding of the effectiveness of mild hypothermia in animal experiments and provides a valuable reference for further research and clinical applications.

## 2. Materials and methods

This meta-analysis followed the Preferred Reporting Items for Systematic Reviews and Meta-Analyses (PRISMA) statement [15]. It has been registered on PROSPERO (CRD42023428314).

### 2.1 Literature search strategy

We searched CNKI, WanFang Data, PubMed, Embase, and Web of Science for literature published up to May 5, 2023. The detailed search strategy is shown in the S1 Appendix in S1 File.

### 2.2 Inclusion criteria

The research we included met the following criteria: (a) Randomized controlled animal experiments published in Chinese or English; (b) rat/mouse experiments; (c) the experimental group underwent mild hypothermia pretreatment and then HIRI; and (d) the control group underwent HIRI at room temperature.

### 2.3 Exclusion criteria

Studies were excluded according to the following criteria: (a) Hepatic ischemia-reperfusion injury due to ischemia in other organs; (b)literature with incomplete data or the inability to obtain the full text; and (c) duplicate data or papers.

### 2.4 Outcome measures

The following outcome measures were examined: (a) alanine aminotransferase (ALT); (b) aspartate aminotransferase (AST); (c) hepatocyte apoptosis rate; (d) histopathological score of liver cells; (e) myeloperoxidase (MPO); (f) superoxide dismutase (SOD); and (g) malondialdehyde (MDA).

## 2.5 Study selection and data extraction

Two researchers (LiJuan Wei and Ke Wei) independently performed literature filtering and data extraction, which was followed by cross-verification. In case of discrepancies, a third researcher (ChunXi Chen) provided consultation or judgment. The filtering process involved the initial removal of duplicate literature, after which the article’s topic and summary were read to assess the relevance of the research content. Subsequently, a detailed reading of the full text was performed to make the final inclusion decision. The extracted information included (a) first author; (b) country and year the study was published; (c) breed, gender, body mass, and number of experimental animals; (d) treatment of the experimental and control groups; (e) content required for risk assessment; and (f) end indicators and measurement data related to the study contents.

### 2.6 Literature bias risk assessment

The SYRCLE animal experiment bias risk assessment tool [16] was used to evaluate the bias risk of the included literature, and two researchers (LiJuan Wei and Ke Wei) independently evaluated the included literature and cross-checked the results.

### 2.7 Statistical analysis

This meta-analysis was performed using RevMan 5.4.1 and Stata 15 software. All of the outcome indicators in this review are continuous and also presented as the mean and standard deviation in the included studies. Considering that some outcome indicators are measured using different outcome measures, we used the standardized mean difference (SMD) and 95% confidence interval (CI) as the effect size. Given that the original study used different species, this study used a random effects model for meta-analysis. The heterogeneity test was performed by Cochran’s Q test and Higgins I^2^ test to judge the heterogenic size for the inclusion of a study. Cochran’s Q test with a p-value less than 0.05 considers heterogeneity problematic, when I^2^<50% was considered to be moderate heterogeneity, I^2^≥50% was thought to be high heterogeneity, subgroup analysis was performed, and the source of heterogeneity was further analyzed. Publication bias in the included studies was determined by examining the symmetry of inverted funnel plots. *P*<0.05 indicates that the difference is statistically significant.

## 3. Results

### 3.1 Literature search process and results

**Fig 1:**
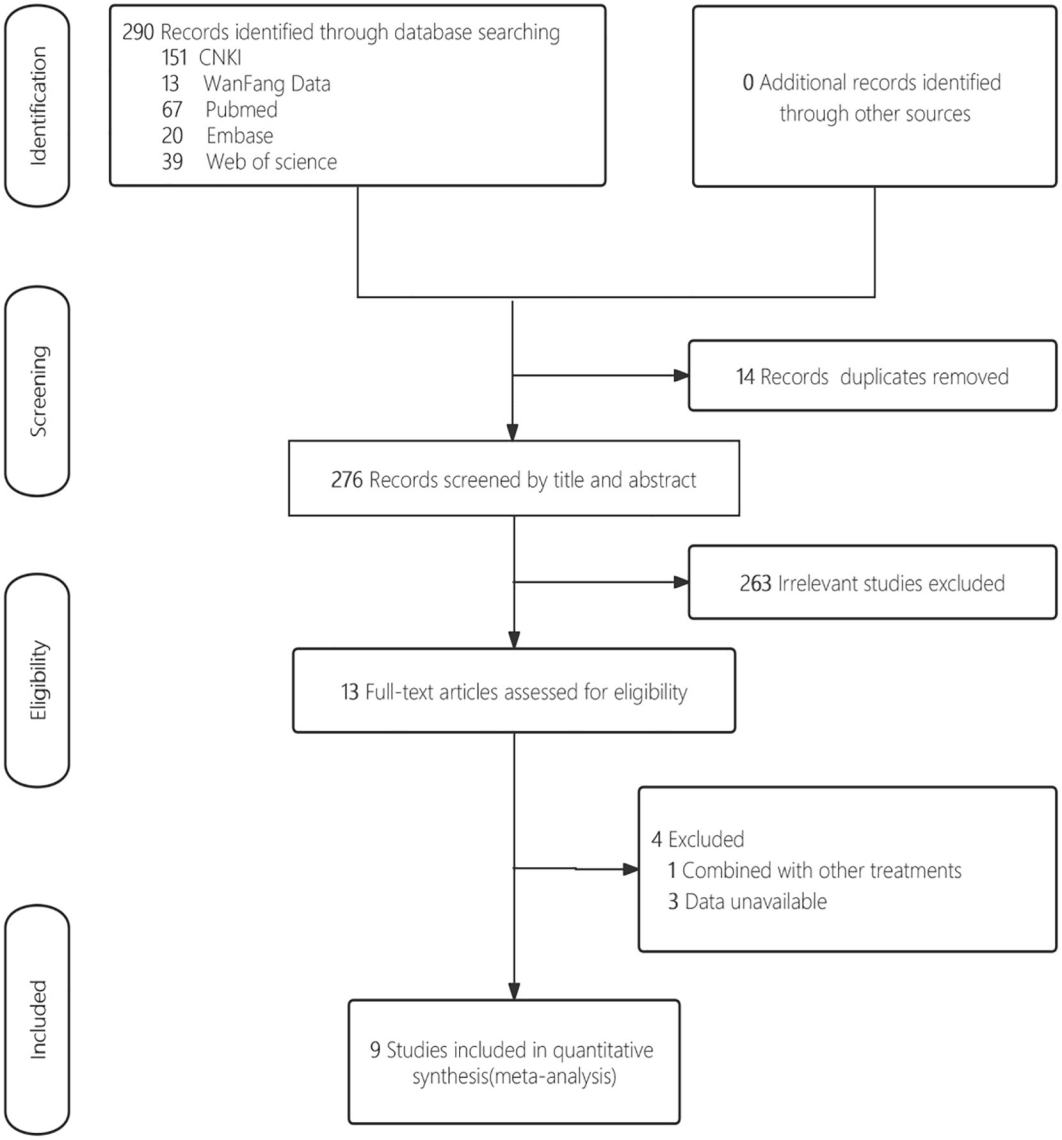
Flowchart of search results and research options.

By searching the databases, a total of 290 related articles were retrieved (151 from CNKI, 13 from WanFang Data, 67 from PubMed, 39 from Web of Science, and 20 from Embase). After eliminating duplicates and after the initial screening, a total of 13 articles were included in the full-text evaluation. 4 studies were excluded after full-text screening, 1 for intervention was combined with other drugs, and 3 for data were unavailable. Ultimately, 9 articles [7, 17–24] were included in this meta-analysis, and a total of 117 rats and mice were included. 59 were pretreated with mild hypothermia, and 58 were subjected to experiments at typical body and room temperatures. Four articles [7, 17, 22, 23] examined mice, and 5 articles [18–21, 24] examined rats. The process and results of the search are shown in Fig 1, and the main characteristics of the included studies are shown in Table 1.

**Table 1:** Included study characteristics.

Author, year	Liu 2021 [17]	Behrend 2006 [18]	Xiao 2017 [19]	Xiao 2022 [7]	Choi 2006 [20]	Wang 2018 [21]	Wang 2020 [22]	Ye 2015 [23]	LIU 2019 [24]
**Country**	China	USA	China	China	Korea	China	China	China	China
**Species/Sex**	C57BL/6N mice /male	Wistar Rat /male	SDRat /male	C57BL/6mice /male	Zucker Rat /male	Sprague-DawleyRat /male	C57BL/6J mice /male	BALB/c mice /male	SDRat /male
**Number of animals (T/C)**	3/3	6/6	8/8	6/6	10/9	6/6	5/5	5/5	10/10
**Duration of ischemia and reperfusion (I/R hours)**	1/6	0.75/24	1/6	1/6	1.25/24	1/6	1/6	0.583/24	1/6
**T (Body temperature after mild hypothermia pretreatment)**	32°C	34°C	32.2±0.5°C	32°C	34°C	32.2±0.5°C	32.2±0.25°C	32.2±0.5°C	32°C
**Preprocessing time (hours)**	NA	remain constant	2	2	remain constant	2	2	2	remain constant
**Whether to warm up after cooling down/temp (°C)**	No	No	Yes /37±0.5	Yes /37	No	No	Yes /36.2±0.2	Yes /37	No /32±0.2
**C**	Modelling at conventional room and body temperatures	Modelling at 37°C body temperature	Modelling of rectal temperature maintained at 37±0.5°C	Core temperature is kept at 37°C for modelling	Modelling of body temperature maintained above 37°C	Modelling to maintain body temperature at 37±0.5°C	Modelling at room temperature	modelling at room temperature	Modelling at conventional room and body temperatures
**Outcome measures**	①②③④	①②④⑤	①③④⑤	①②③④⑥⑦	①②	①③④	①②③④⑥⑦	①②③	①②⑥⑦

T: experimental group; C: control group; NA: Not Applicable; ①ALT; ②AST; ③Hepatocyte apoptosis rate; ④Hepatocyte pathology score; ⑤MPO; ⑥SOD; ⑦MDA。

### 3.2 Study quality

The risk of bias for the articles is shown in Table 2. Overall, all studies are at high risk of bias in at least one domain, mainly focused on blinding of researchers and caregivers and blinding of outcome assessment. Taking into account the heterogeneity that exists, the risk of bias, and the publication bias that has been identified, two researchers(LiJuan Wei and Ke Wei) assessed the certainty of the evidence (also known as the quality of the evidence) using the Grading of Recommendations Assessment, Development, and Evaluation (GRADE) approach at the outcome level. The included studies were all randomized controlled trials, which could have begun as evidence of high certainty, but the certainty was reduced due to large biases in randomization, allocation concealment and blinding, small sample sizes for inclusion, and publication bias(S2 Appendix in S1 File).

**Table 2:** Risk of bias evaluation of included studies.

Author, year	Country	①	②	③	④	⑤	⑥	⑦	⑧	⑨	⑩
**Ye 2015** [21]	China	Unclear	Low	Unclear	Unclear	High	Unclear	High	Low	Low	Unclear
**Wang 2018** [19]	China	Unclear	Low	Unclear	Low	High	Unclear	High	Low	Low	Unclear
**Xiao 2017** [17]	China	Unclear	Low	Unclear	Low	High	Unclear	High	Low	Low	Unclear
**Wang 2020** [20]	China	Unclear	Low	Unclear	Low	High	Unclear	High	Low	Low	Unclear
**Liu 2021** [15]	China	Unclear	Low	Unclear	Low	High	Unclear	High	Low	Low	Unclear
**Xiao 2022** [5]	China	Unclear	Low	Unclear	Low	High	Unclear	High	Low	Low	Unclear
**Behrends 2006** [16]	USA	Unclear	Low	Unclear	Low	High	Unclear	High	Low	Low	Unclear
**Choi 2005** [18]	Korea	Unclear	Low	Unclear	Low	High	Unclear	High	Low	Low	Unclear
**LIU 2019** [24]	China	Unclear	Low	Unclear	Low	High	Low	High	Low	Low	Unclear

①Random sequence generation; ②Baseline characteristics; ③Allocation concealment; ④Random housing of animals; ⑤Blinding of researchers and caregivers; ⑥Random outcome assessment; ⑦blinding of outcome assessment; ⑧Incomplete outcome data; ⑨Selective outcome reporting; ⑩Other sources of bias.

### 3.3 Effect of mild hypothermia pretreatment on liver function after HIRI

#### 3.3.1 ALT and AST

**Fig 2:**
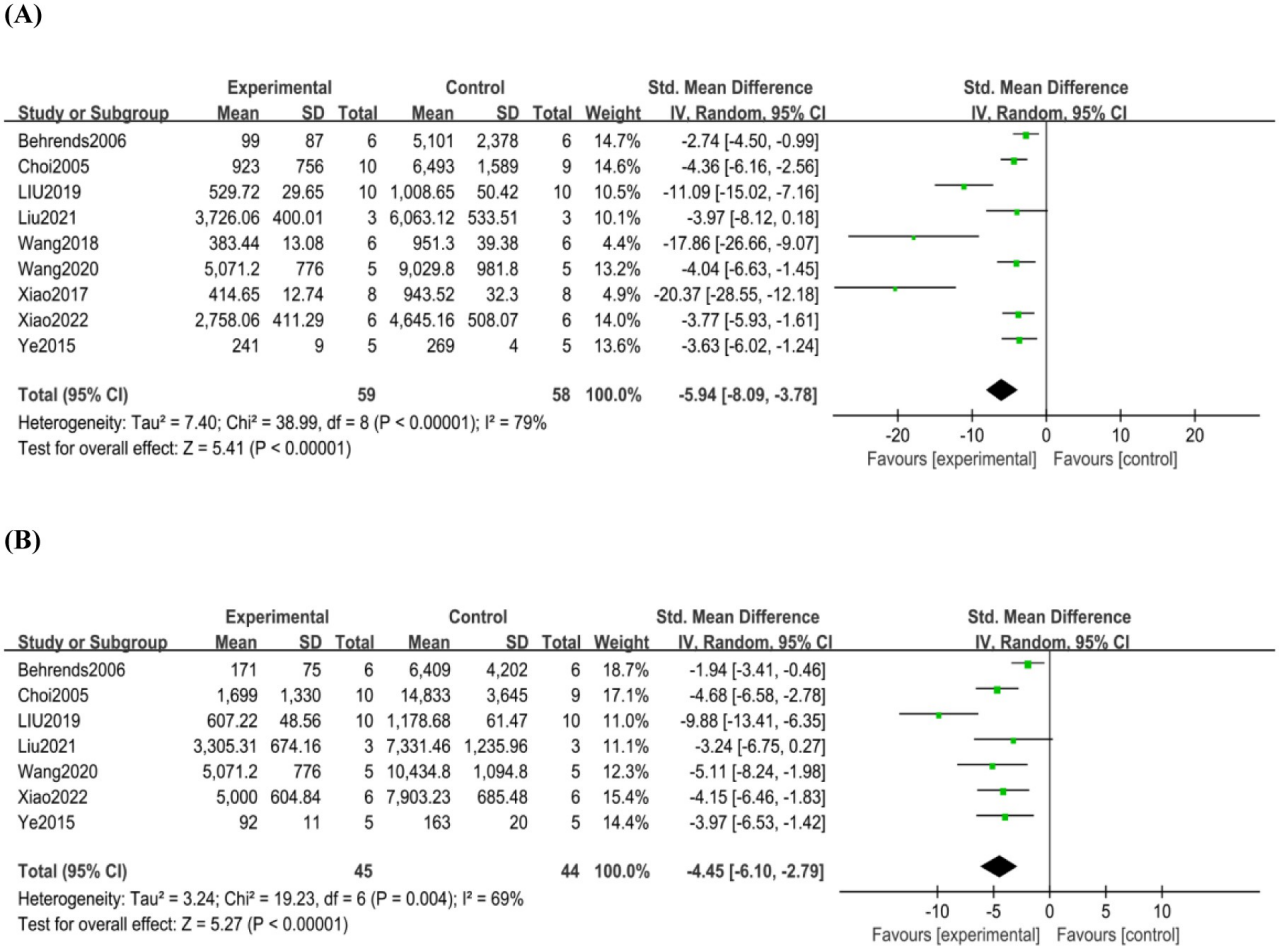
(A)Analysis of the ALT, (B)Analysis of the AST.

The nine included studies [7, 17–22, 24] all reported the effect of mild hypothermia pretreatment on ALT levels in one hundred and seventeen rats/mice. In three of the studies [18, 20, 23], ALT was measured in blood collected 24 hours after reperfusion, in five studies [7, 17, 19, 21, 22], ALT was measured in blood collected 6 hours after reperfusion, and in one study [24] collected 12 hours after reperfusion. Seven studies [7, 17, 18, 20, 22–24] reported the effect of subfreezing temperatures on AST levels in eighty-nine rats/mice. In three of the studies [18, 20, 23], AST was measured by blood collected 24 hours after reperfusion, in three studies [7, 17, 22], AST was measured by blood collected 6 hours after reperfusion, and in one study [24] collected 12 hours after reperfusion. ALT and AST levels were significantly reduced in the mild hypothermia pretreatment group [ALT: SMD -5.94, 95% CI -8.09 to -3.78 (Fig 2A); AST: SMD -4.45, 95% CI -6.10 to -2.78 (Fig 2B)]. And our analyses also showed high heterogeneity(ALT: *p*<0.01, I^2^ = 79%; AST: *p*<0.01, I^2^ = 69%).

So we performed subgroup analyses of ALT levels by species, length of reperfusion, and pretreatment temperature. The results did not show any directional changes, but the heterogeneity was significantly reduced (S3 Appendix in S1 File). To find the source of heterogeneity, we performed sensitivity analyses of ALT levels and compared the results by species, duration of reperfusion, and pretreatment temperature. The combined effect sizes were within the 95% confidence intervals when any of the studies were excluded, suggesting that the differences in species, duration of reperfusion, and pretreatment temperature did not affect the stability of the results after combining the studies. (S4 Appendix in S1 File).

We created an inverted funnel plot of ALT levels, and the results showed that the two sides were not symmetrical; we concluded that there may be publication bias in the included articles (S5 Appendix in S1 File).

### 3.4 Effect of mild hypothermia on liver cells and tissues after HIRI

#### 3.4.1 Hepatocyte apoptosis

**Fig 3:**
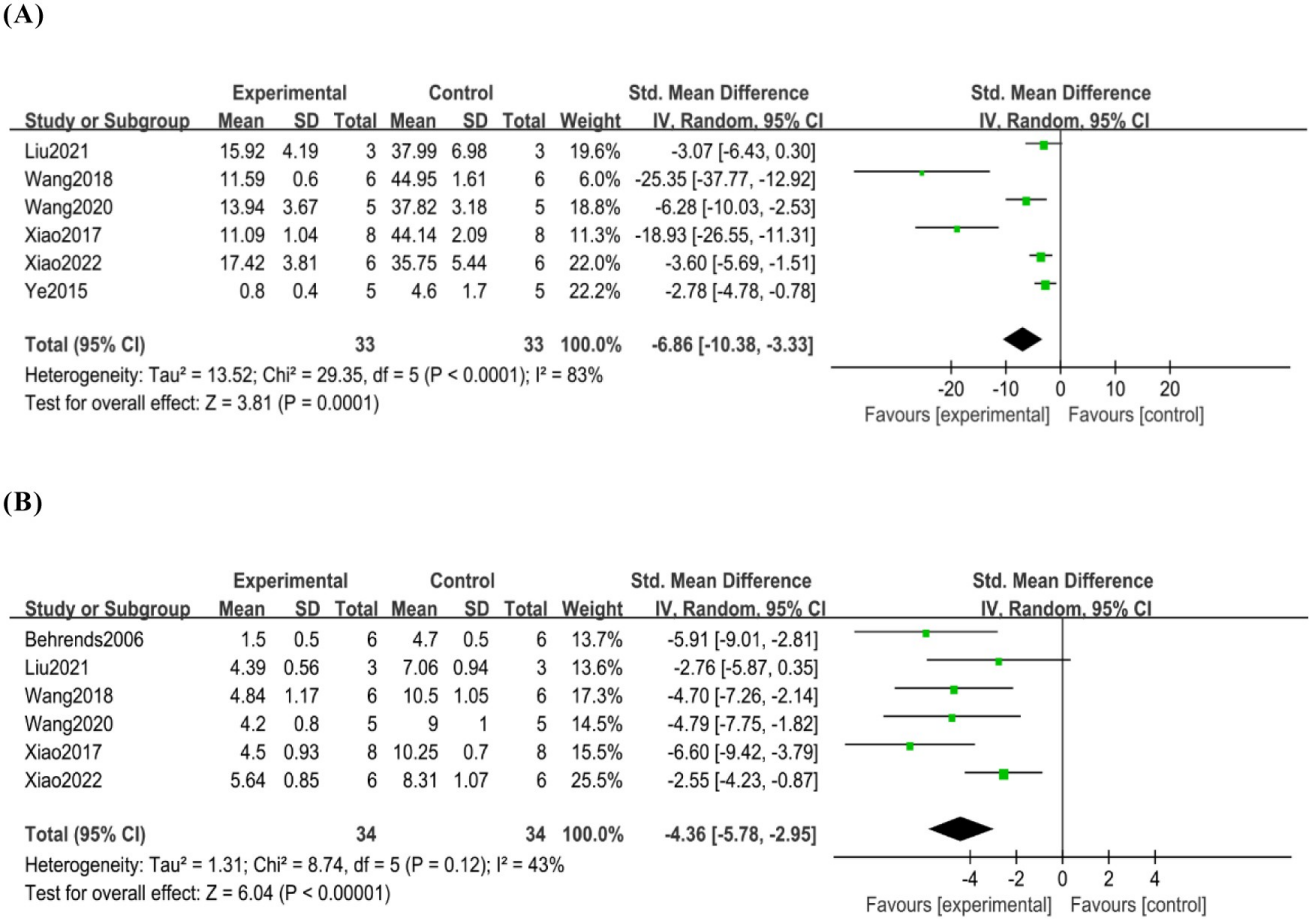
(A)Analysis of hepatocyte apoptosis, (B)Analysis of the level of liver injury after HIRI.

Six studies [7, 17–19, 21, 22] reported the effect of mild hypothermia on the level of liver injury in HIRI in thirty-three rats/mice in the mild hypothermia group and thirty-three rats/mice in the normothermic control group. In these studies, tissue samples were obtained after reperfusion, and hepatocyte apoptosis was detected by TUNEL assay. Mild hypothermia significantly reduced hepatocyte apoptosis after HIRI (SMD -6.86, 95% CI -10.38 to -3.33) (Fig 3A). Our analyses also showed high heterogeneity (*p*<0.01, I^2^ = 83%).

#### 3.4.2 Hepatocyte pathology score

Six studies [7, 17–19, 21, 22] reported the effect of mild hypothermia on the level of liver injury after HIRI in thirty-four rats/mice in the mild hypothermia group and thirty-four rats/mice in the normothermic control group. In these studies, tissue samples were obtained after reperfusion. Except for the study by Behrends et al. [18] which used the Histological Score, the rest of the studies used the Suzuki standard to determine the severity of hepatic IR injury. However, in both scoring methods, a higher score means more severe liver injury. Mild hypothermia pretreatment significantly reduced the level of liver injury after HIRI (SMD -4.36, 95% CI -5.78 to -2.95) (Fig 3B). Our analyses also showed moderate heterogeneity (*p* = 0.12, I^2^ = 43%).

### 3.5 Effect of mild hypothermia on levels of inflammation and oxidative stress following HIRI

#### 3.5.1 Myeloperoxidase (MPO) levels

**Fig 4:**
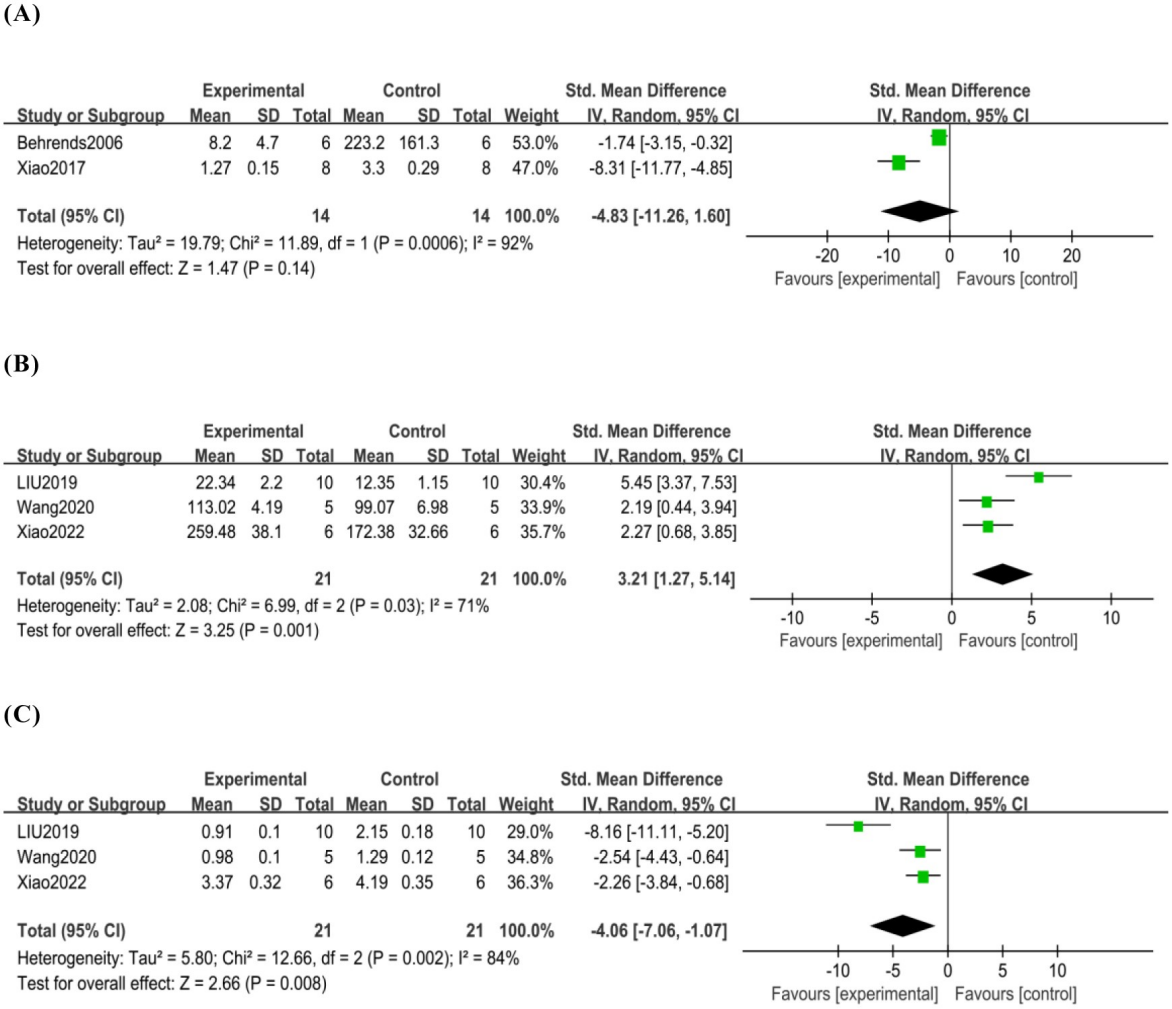
(A)Analysis of the MPO, (B)Analysis of SOD, (C)Analysis of MDA.

MPO assay determines hepatic neutrophil activity. Two studies [18, 19] reported the effect of mild hypothermia on MPO levels in HIRI; fourteen rats/mice underwent mild hypothermia and fourteen rats/mice were in the normothermic control group. Tissue samples were obtained after reperfusion, but the units of measurement used in the two studies were different. There was no significant difference in MPO levels between the mild hypothermia and normothermic control groups (SMD -4.83, 95% CI -11.26 to 1.60) (Fig 4A). Our analyses also showed high heterogeneity (*p*<0.01, I^2^ = 92%).

#### 3.5.2 Oxidative stress levels

Three studies [7, 22] reported the effects of mild hypothermia pretreatment on SOD levels and MDA levels, and the experimental and control groups each included twenty-two rats/mice. All tissue samples were taken after the end of reperfusion and tested using a colorimetric assay kit. Mild hypothermia pretreatment significantly increased SOD levels (SMD 3.21, 95% CI 1.27 to 5.14) (Fig 4B) and decreased MDA levels (SMD -4.06, 95% CI -7.06 to -1.07) (Fig 4C) after HIRI. Our analyses also showed high heterogeneity(SOD: *p* = 0.03, I^2^ = 71%; MDA: *p*<0.01, I^2^ = 84%).

## 4. Discussion

The results of our meta-analysis showed that mild hypothermic preconditioning was effective in reducing hepatic ischemia-reperfusion injury. However, the results of our analyses should be interpreted with caution due to the small number of studies in this area, the low quality of the studies, and the exclusion of one conference abstract [25] without detailed data. Although mild hypothermia improved the vast majority of outcome indicators, it was associated with high statistical heterogeneity (I^2^ = 43–92%) and studies showed significant bias and quality issues. Three studies compared more than two groups. Behrends et al. [18] conducted a study on the effects of moderate hypothermia (31°C) and mild hypothermia (34°C) on HIRI. Because the temperature range we chose was considered to be mildly hypothermic, we chose the 34°C treatment as an indicator of outcome. The experimental subjects in the study by Choi et al. [20] were obese and lean rats, but only three rats in the obese group survived after HIRI. In comparison, nine rats in the lean group survived, and considering that the health status of lean rats is closer to that of normal rats, we included the outcome metrics of lean rats in our analyses. Jian Liu et al. [24] grouped different reperfusion times in their study, and since the most severe damage occurred at 12 hours of reperfusion, the authors also chose the node of 12 hours for liver tissue to be tested for apoptotic proteins and oxidative stress and other markers, and therefore we included the outcome indicators for the time point of 12 hours of reperfusion. These would lead to a reduction in our confidence in the current literature in predicting whether mild hypothermia affects HIRI outcomes and the magnitude of its effect. More data from high-performance validation studies are needed to understand the true amount of effect better before further clinical studies can be conducted.

HIRI is a complex and inevitable pathological process that develops during the entire course of donor organ procurement, preservation, and liver transplantation. Severe HIRI in liver transplantation can lead to acute or chronic rejection, and it can even result in graft failure due to the induction of inflammation and oxidative stress [6, 26]. The main causes of liver injury during ischemia-reperfusion are reactive oxygen species (ROS), apoptosis, and the inflammatory response, as reported in previous studies [27, 28]. Several studies have confirmed the involvement of various factors, such as intracellular calcium overload, Kupffer cells, complement, noncoding RNAs, and autophagy, in the mechanism of HIRI [29]. The continuous exploration of these mechanisms may be an important reference for improving HIRI.

As with the results of our meta-analysis, previous studies have demonstrated the positive role of mild hypothermia in IRI. Previous studies have shown that mild hypothermia regulates cellular free radical production, calcium content, and pH during the initial phase of ischaemic injury. Furthermore, during the reperfusion injury period after the restoration of the blood supply, mild hypothermia modulates downstream necrotic, apoptotic, and inflammatory pathways, thereby delaying cell death [30]. Mild hypothermia at 32°C represents the optimal overlap between activating and inhibiting mechanisms, and the protective effect of mild hypothermia against IRI may be attributed to metabolic inhibition and enhanced stress tolerance, ultimately reducing stress [31]. Recent studies have suggested that the protective mechanism by which mild hypothermia can treat HIRI involves mitochondrial metabolism, immune regulation, glucose metabolism, and transporter proteins. The results of our meta-analysis indicate that mild hypothermic preconditioning downregulates ALT levels and AST levels and attenuates hepatocyte apoptosis, the inflammatory response, the degree of liver tissue damage, and oxidative stress, effectively protecting liver tissue and might attenuate IRI in liver transplantation, based on evidence from animal studies. In agreement with Longo et al. [32], who believed that mild hypothermia pretreatment yielded better outcomes than ischaemic preconditioning. Mechanistically, hypothermia reduces hepatic oxygen consumption during ischemia, attenuates endothelial injury and microcirculatory disturbances during reperfusion, improves hepatic tissue oxygenation, and maintains hepatocytes in a relatively favorable state, thereby reducing hepatocellular injury after reperfusion [33, 34].

However it is essential to consider that core hypothermia during clinical liver transplantation may directly impair the patient’s immune function and trigger thermoregulatory vasoconstriction, which can lead to reduced oxygen supply to the wound, delayed wound healing, and infection risks [35]. For every 1 degree Celsius drop in core body temperature, the risk of bleeding increases by about 20% [36], and coagulation is essential during clinical liver transplantation. Consequently, the use of thermal insulation measures remains necessary to prevent patient hypothermia during clinical liver transplantation. It can be seen that lowering the patient’s body temperature to 32–34°C preoperatively is not practical at this time. However, mild hypothermia may be used in liver preservation and machine perfusion, where it is more effective than deep hypothermia in protecting the organ and reducing the complications of hypothermia resulting from liver implantation into the recipient. It has been demonstrated that organ donor hypothermia of 34–35°C significantly reduces the incidence of delayed recovery of recipient graft function and improves 1-year graft survival, whereas mild hypothermia does not adversely affect donor physiology or extrarenal graft survival [37, 38]. Ex-situ hypothermic oxygenated machine perfusion (HOPE) is a well-established practice in liver transplantation, which is used for liver resuscitation in liver transplantation, and its beneficial effects in protecting mitochondria from initial damage, improving complex I-V function, replenishing ATP, and preparing for normothermic reperfusion during organ transplantation, and protecting the biliary tree from major damage are undeniable [39, 40]. But Martins et al. [41] also found that machine perfusion temperature at 32°C provided more effective protection of the liver and reduced possible coagulation disorders associated with hypothermic machine perfusion. Further functional and technical studies are needed to integrate it into clinical practice and apply it to liver transplantation.

The activation of organ-protective substances induced by mild hypothermia could potentially be used in clinical liver transplantation and offer a strategy to minimize HIRI during transplantation and improve the survival of transplanted livers. In the mild hypothermia state, activation of the NF-κB p65 pathway resulted in high expression of the RNA-binding protein RBM3, which effectively prevented apoptosis [42]. Autophagy also plays a significant role in IR injury, and the activation of autophagic flux contributes to IR injury tolerance; mild hypothermia reduces AP accumulation and restores autophagic flux through AP-lysosomal fusion mediated by the Rab7-SNARE pathway [17, 43]. Additionally, mild hypothermia protects mitochondrial function, corrects lipid metabolism, preserves hepatic FAO activity, and attenuates lipid peroxidation and oxidative stress damage during IR by activating the JAK2/STAT3 pathway [22, 44, 45]. These findings provide some support for the results of the meta-analysis. It’s worth noting that as an indicator of neutrophil accumulation, MPO activity did not show a significant difference in the combined analysis. This result contradicts the findings of the two studies included in the analysis and several other studies demonstrating the effectiveness of mild hypothermia in reducing MPO levels after organ ischemia‒reperfusion [46, 47]. MPO is a measure of hepatic neutrophil activity that reflects aseptic inflammation due to macrophage activation and neutrophil recruitment triggered by liver IR [48]. This discrepancy may be attributed to the difference in the units used to indicate MPO levels in the two included studies. Moreover, this result only combines two studies, and the GRADE level of evidence rating is very low, which shows that there is still a lack of evidence. The same is true for the results of SOD and MDA, which, due to the small number of included studies, still suffer from a lack of evidence even if they are statistically significant. Additionally, the experimental rats and mice in all studies were not subjected to prolonged postoperative observation and follow-up, and the long-term effects of mild hypothermia on postoperative recovery and the function of the transplanted organs have yet to be determined.

Despite this retrospective study demonstrating the potential of mild hypothermia for the treatment of HIRI, certain limitations need to be acknowledged. First, there is a lack of studies on mild hypothermia for treating hepatic ischemia-reperfusion injury, particularly randomized controlled trials, and there have not yet been clinical trials, leading to a limited number of included studies, all of which involved animal experiments. Second, our literature search was limited to studies published in English and Chinese and potentially excluded relevant studies in other languages that met our criteria. Third, the inconsistent baseline level of animals in the included studies might have reduced the comparability of some outcomes. Fourth, despite conducting subgroup analyses, full resolution of heterogeneity was not achieved, which might have influenced the accuracy of the results.

In conclusion, our study indicates that mild hypothermia can attenuate hepatic ischemia-reperfusion injury, effectively reducing oxidative stress and the inflammatory response, preventing hepatocyte apoptosis, and protecting liver function. Given the limitations in the quality and quantity of the included literature, there is still a lack of overall evidence on the topic, further high-quality randomized controlled studies are required to validate the findings of this study.

## Supporting information

S1 Checklist PRISMA 2020 checklist. (DOCX)

S1 Data (RAR)

S1 File (DOCX)
