# Supplementary material for: Mild hypothermia pretreatment improves hepatic ischemia-reperfusion injury: A systematic review and meta-analysis of animal experiments
Source: PLoS One. 2024 Jul 2;19(7):e0305213. doi: 10.1371/journal.pone.0305213 (PMC11218962; doi:10.1371/journal.pone.0305213)
Supplement: S1 File — (DOCX) [file pone.0305213.s003.docx]

| search strategy |
| --- |
| #1 Hypothermia, Induced[Mesh]  #2 Therapeutic Hypothermia OR Hypothermia, Therapeutic OR Targeted Temperature Management OR Targeted Temperature Managements OR Induced Hypothermia OR Moderate Hypothermia, Induced OR Induced Moderate Hypothermia OR Induced Moderate Hypothermias OR Moderate Hypothermias, Induced OR Mild Hypothermia, Induced OR Induced Mild Hypothermia OR Induced Mild Hypothermias OR Mild Hypothermias, Induced) [All Fields]  #3 #1 OR #2  #4 Hepatic ischemia reperfusion injury[Mesh]  #5 Hepatic ischemia reperfusion injury[All Fields]  #6 #4 OR #5  #7 #3 AND #6 |

**S1. Search strategy**


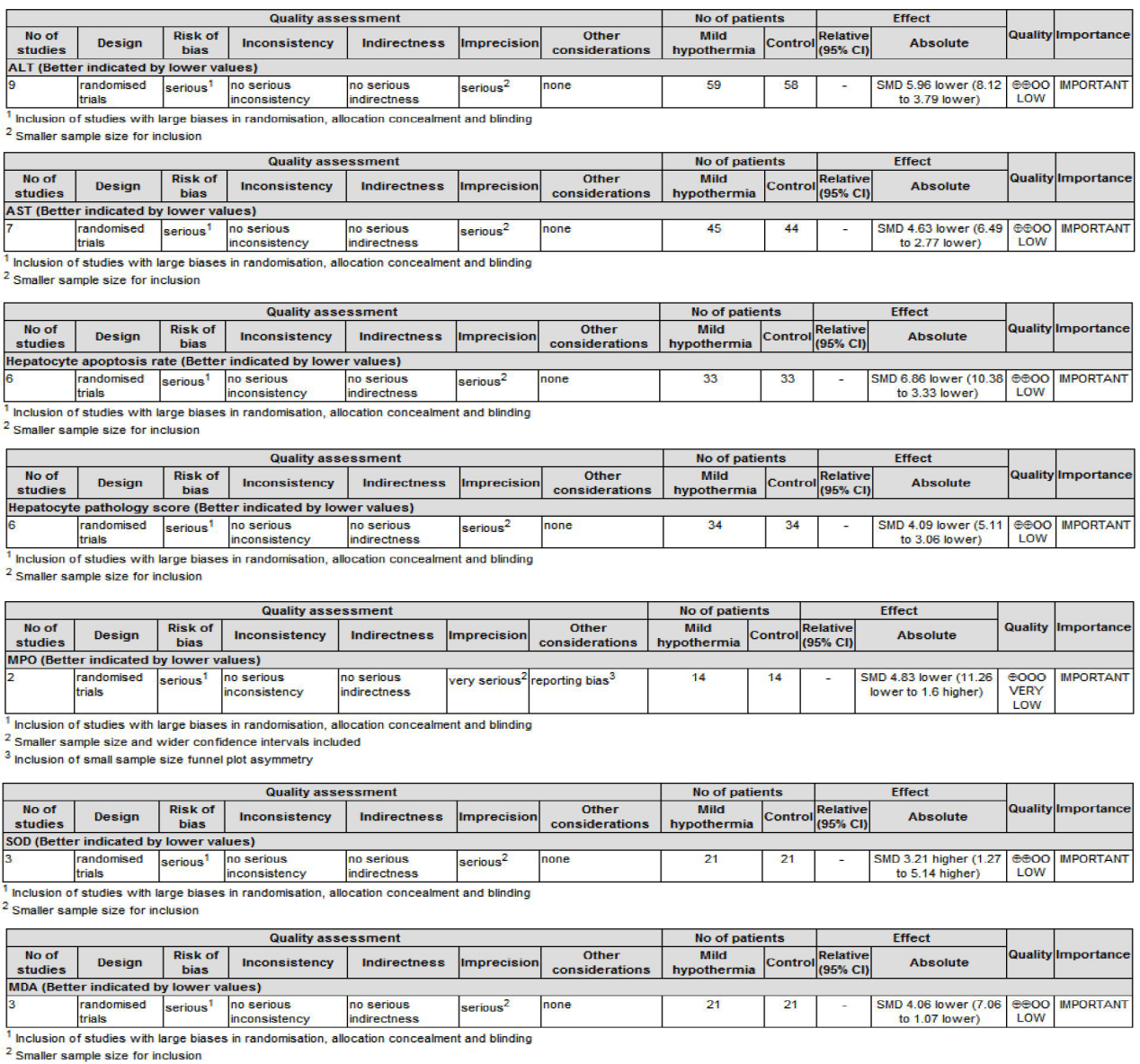


**S2. GRADE evidence rating evaluation**

**
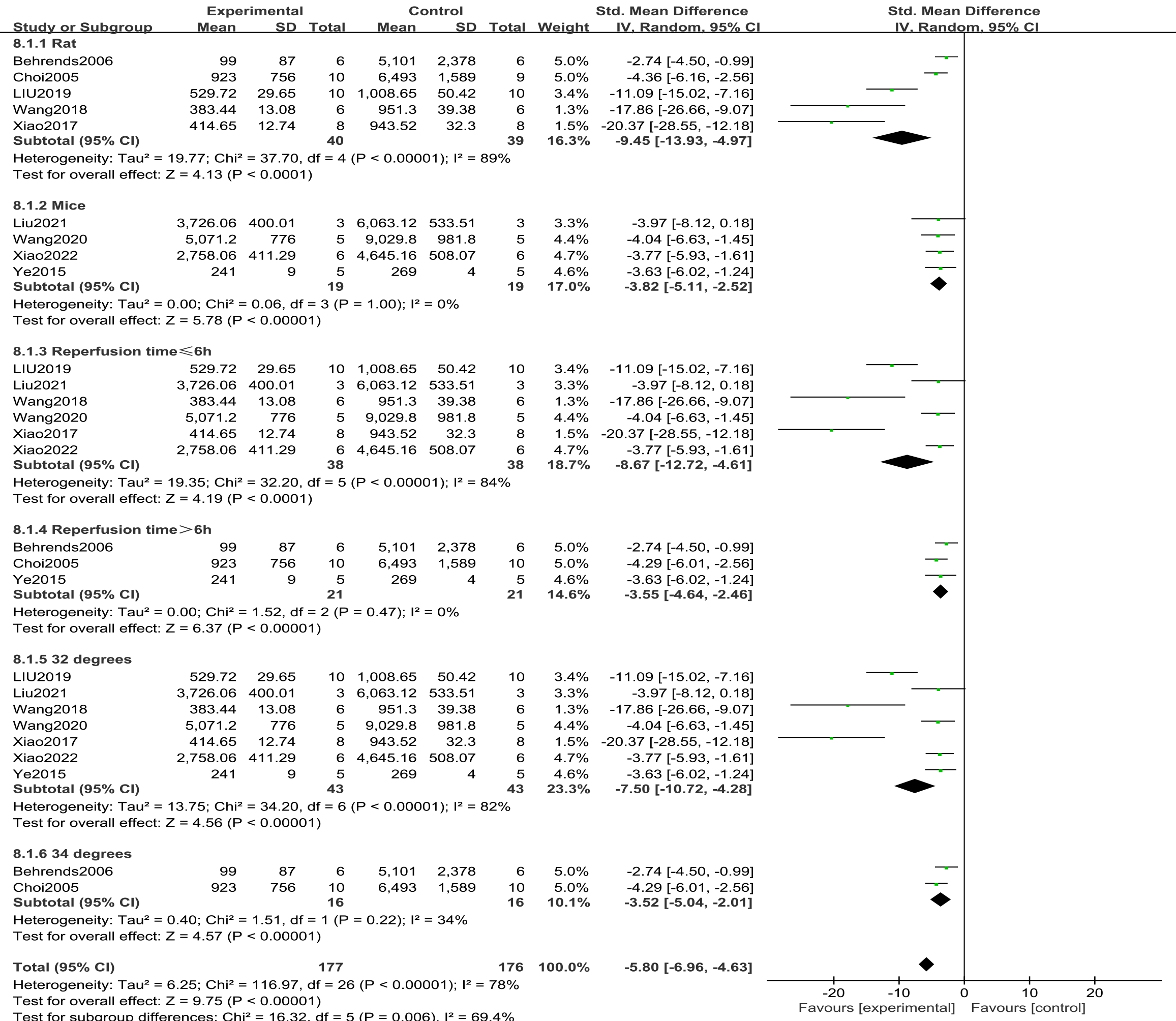

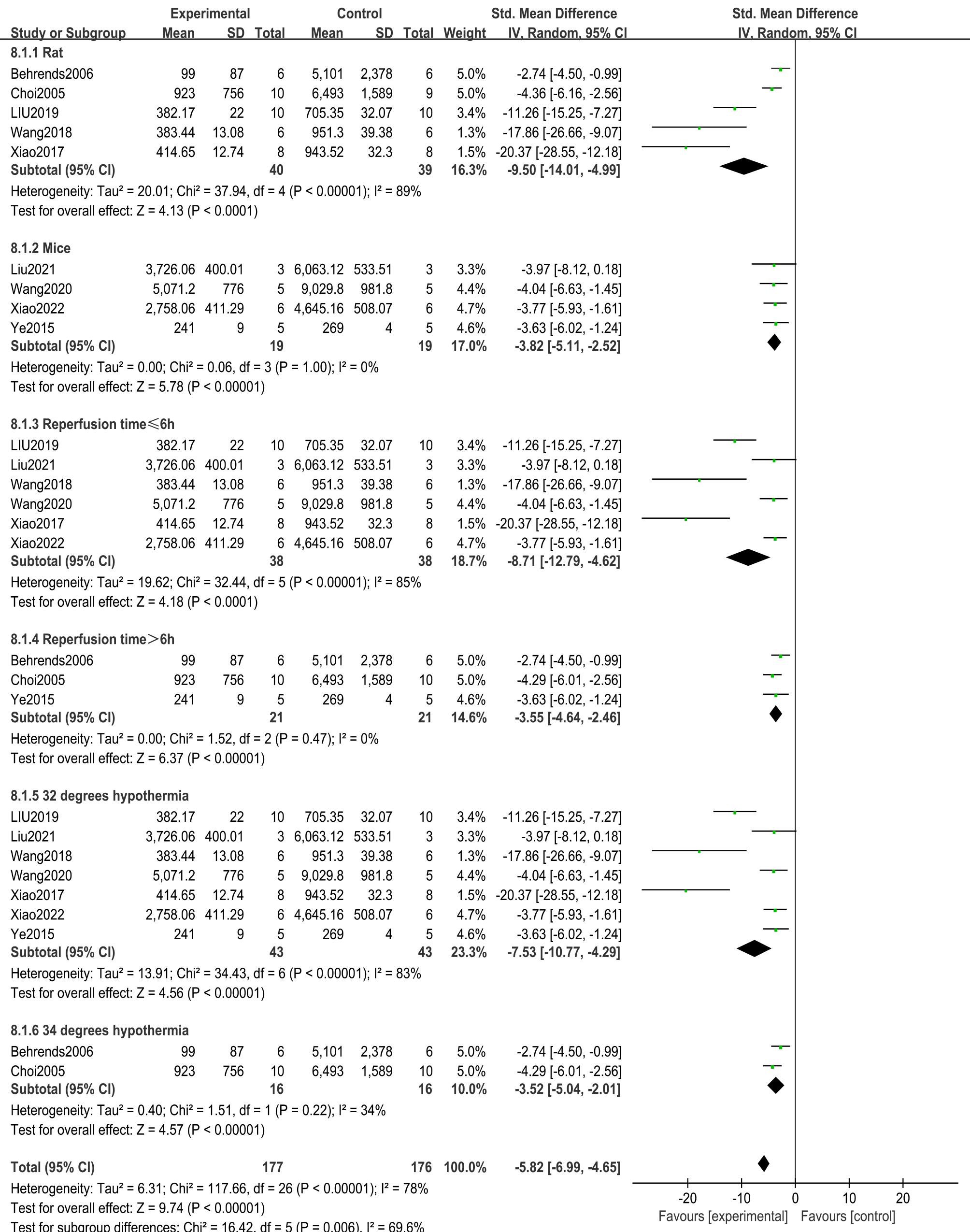
**

**S3. Subgroup analyses of ALT**

**(A)**


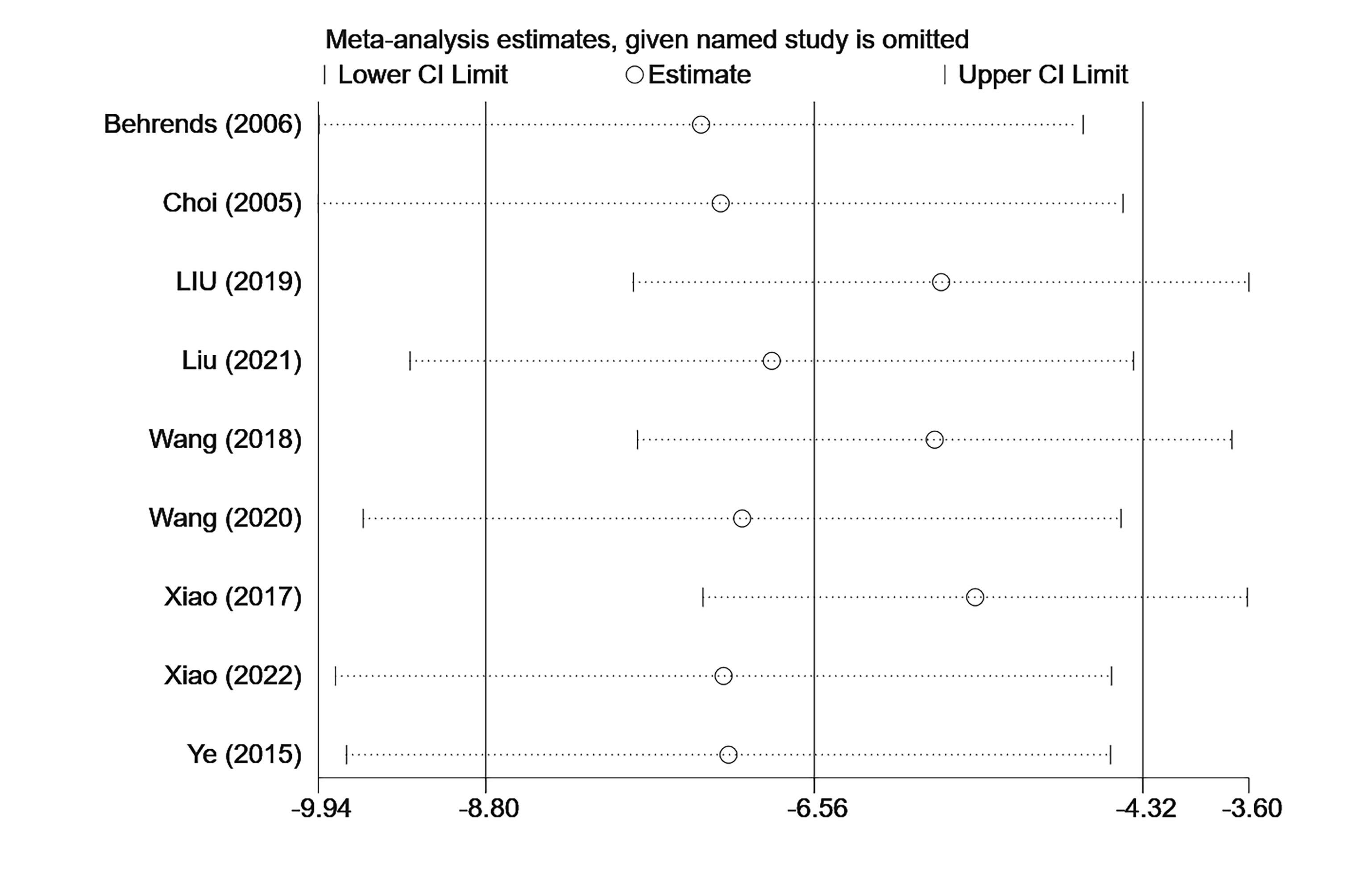


**(B)**


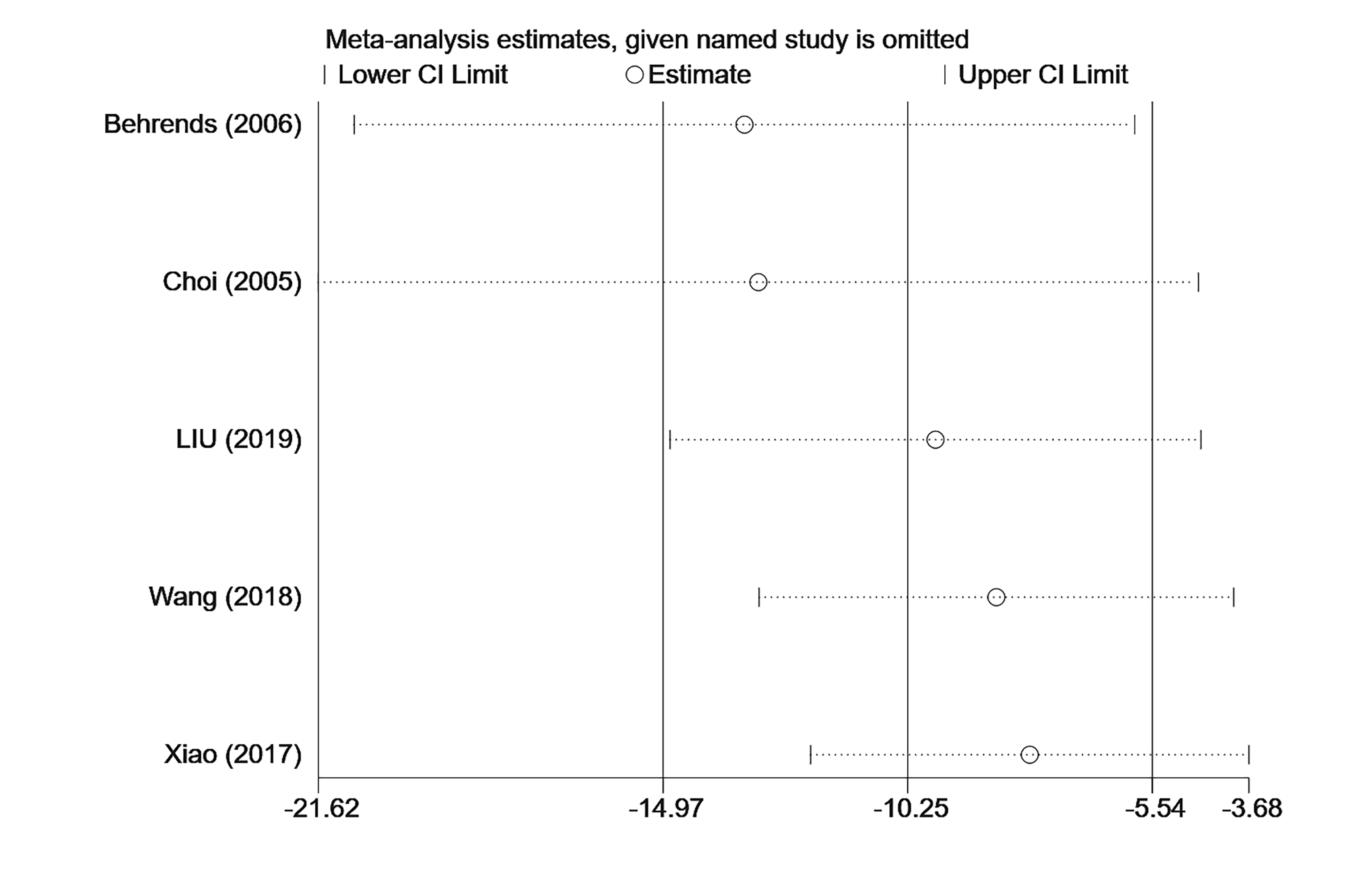


**(C)**

**
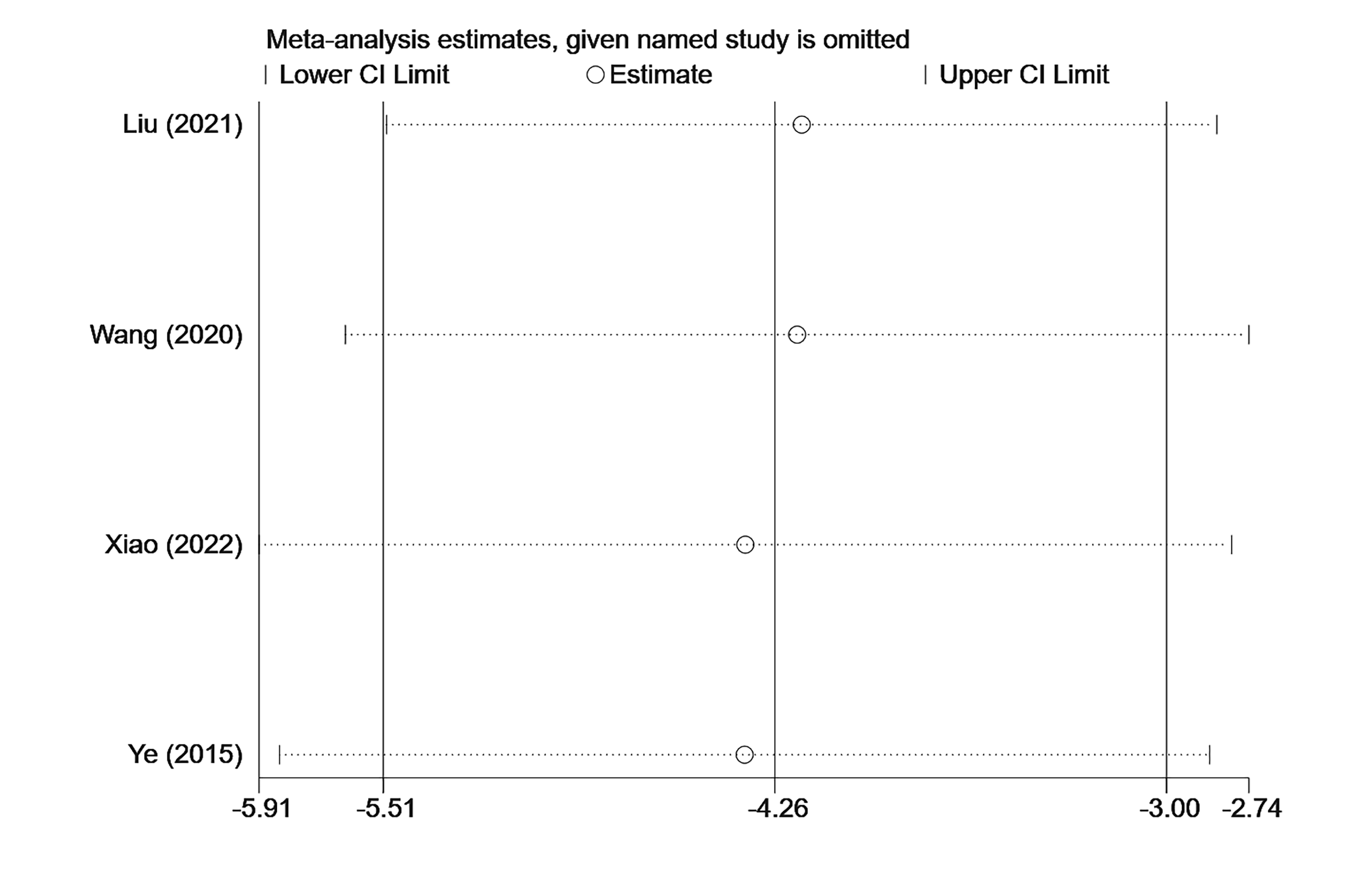
**

**(D)**

**
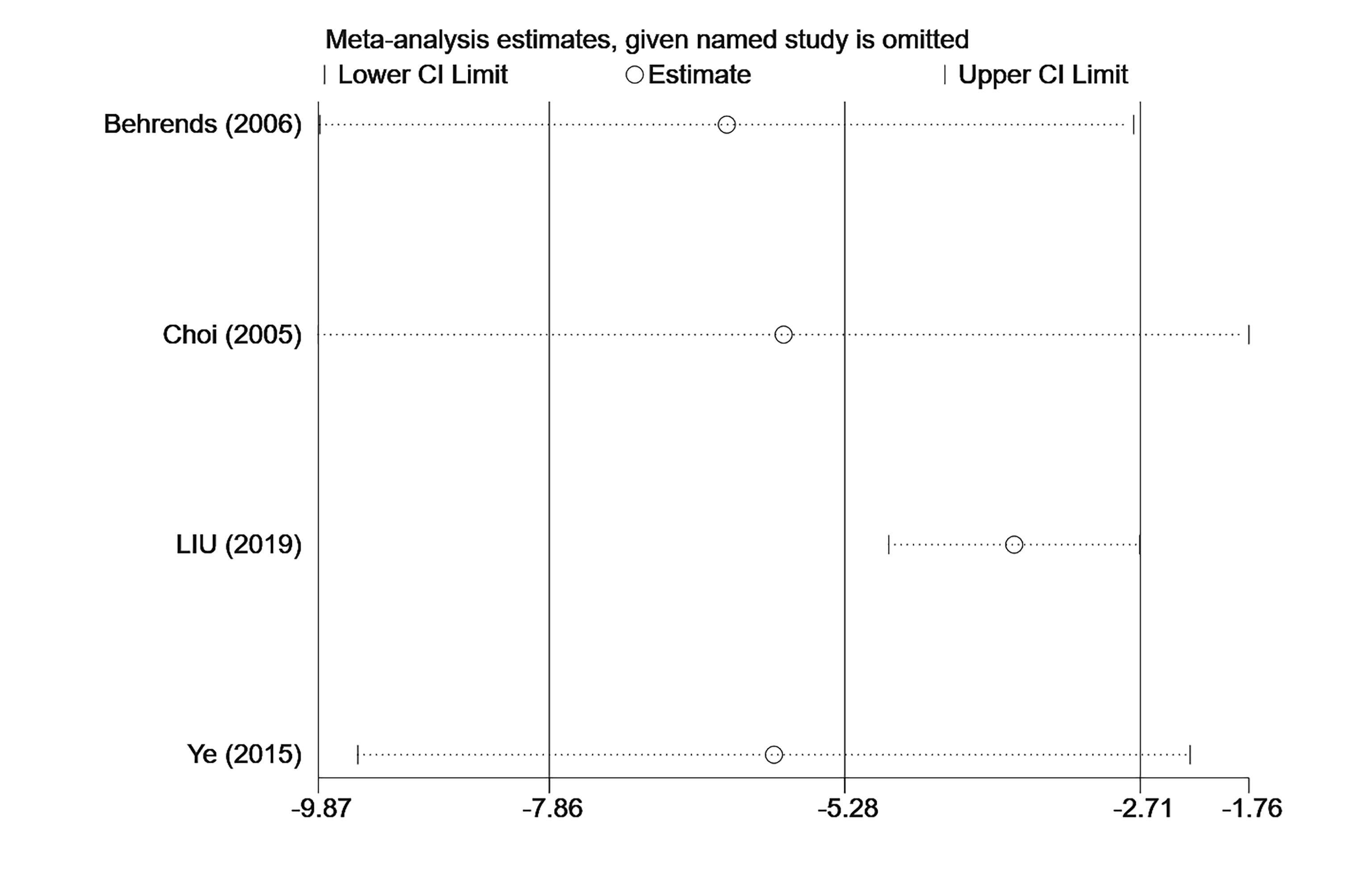
**

**(E)**

**
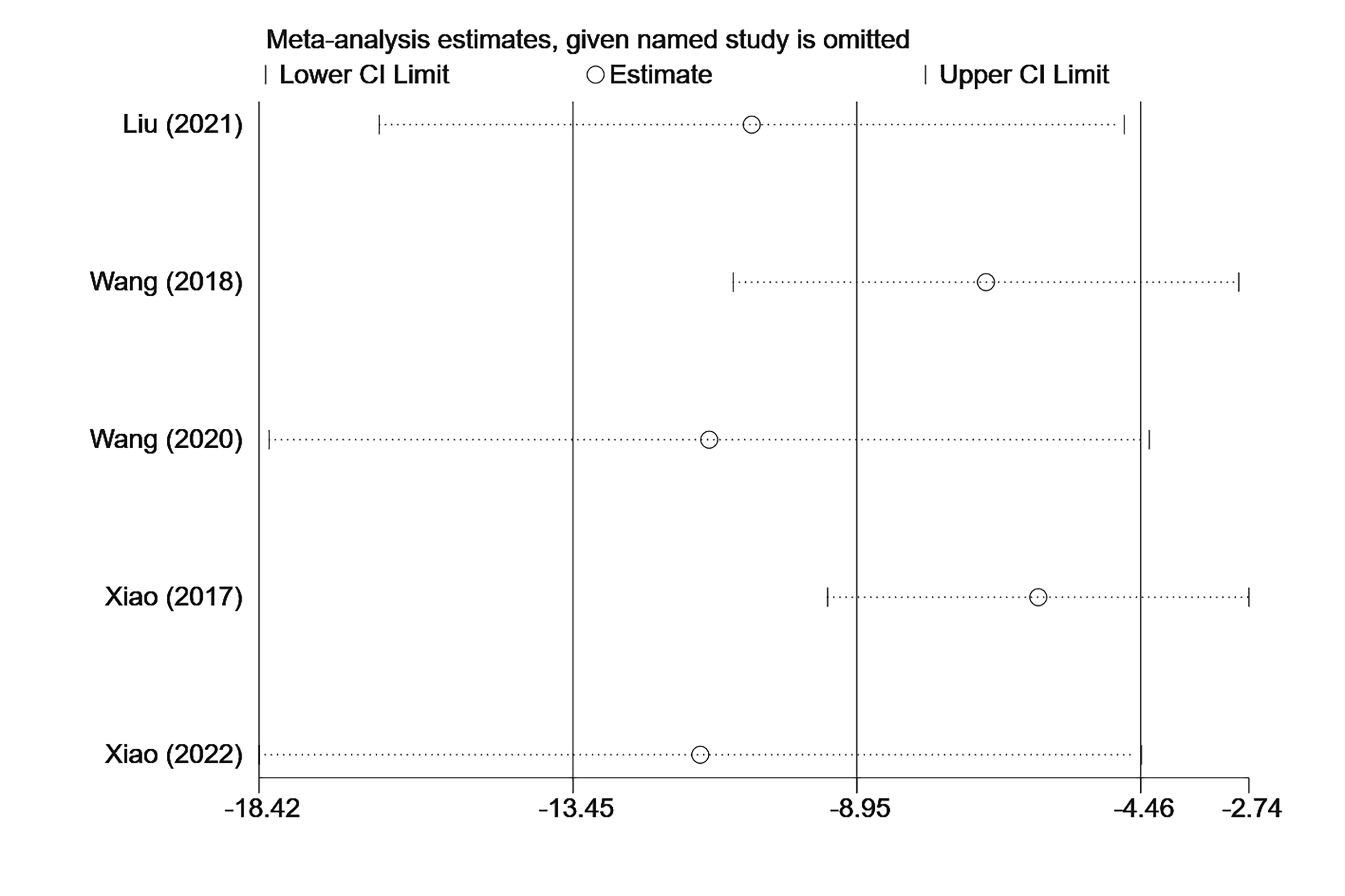
**

**(F)**

**
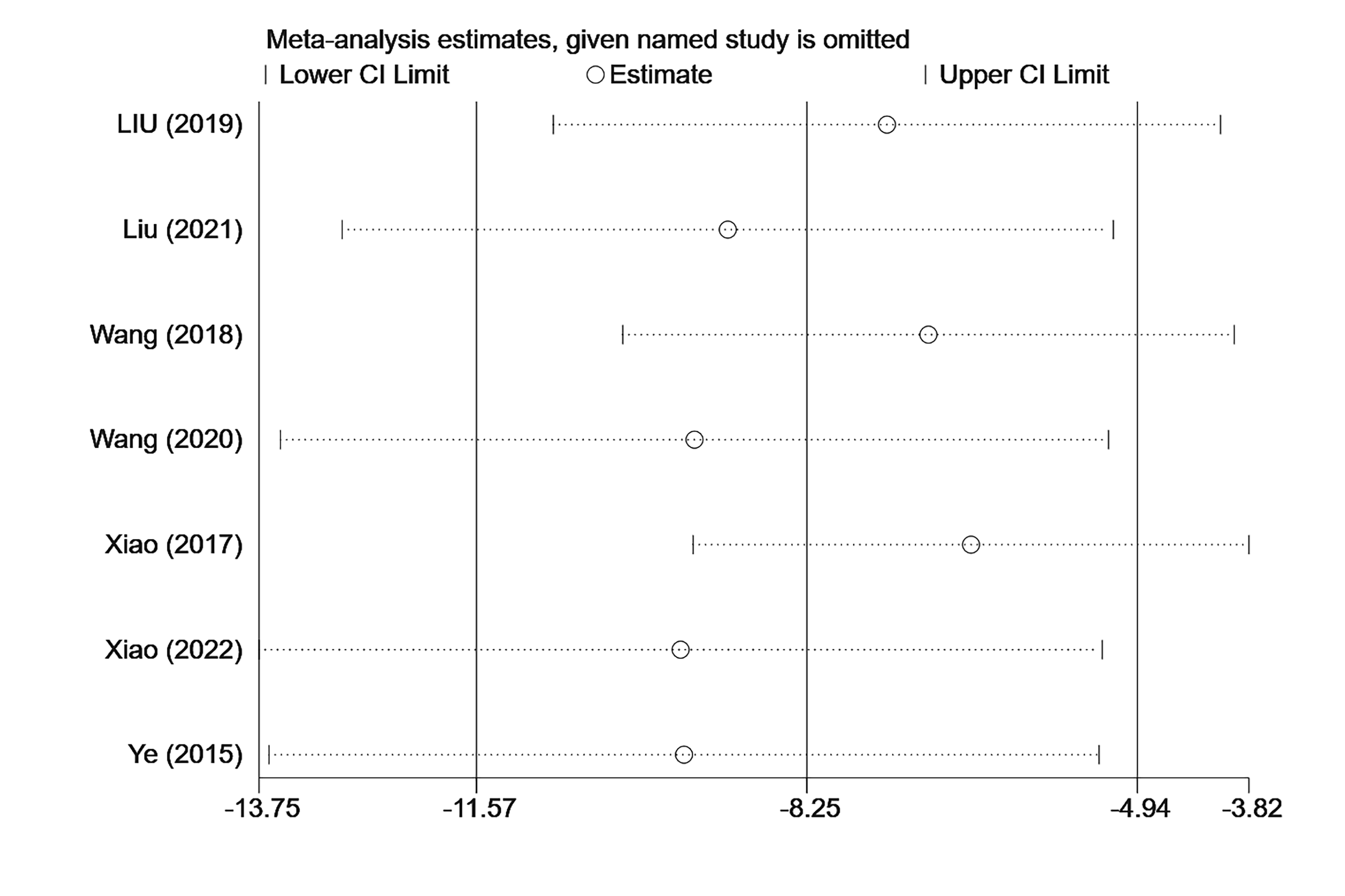
**

**(G)**

**
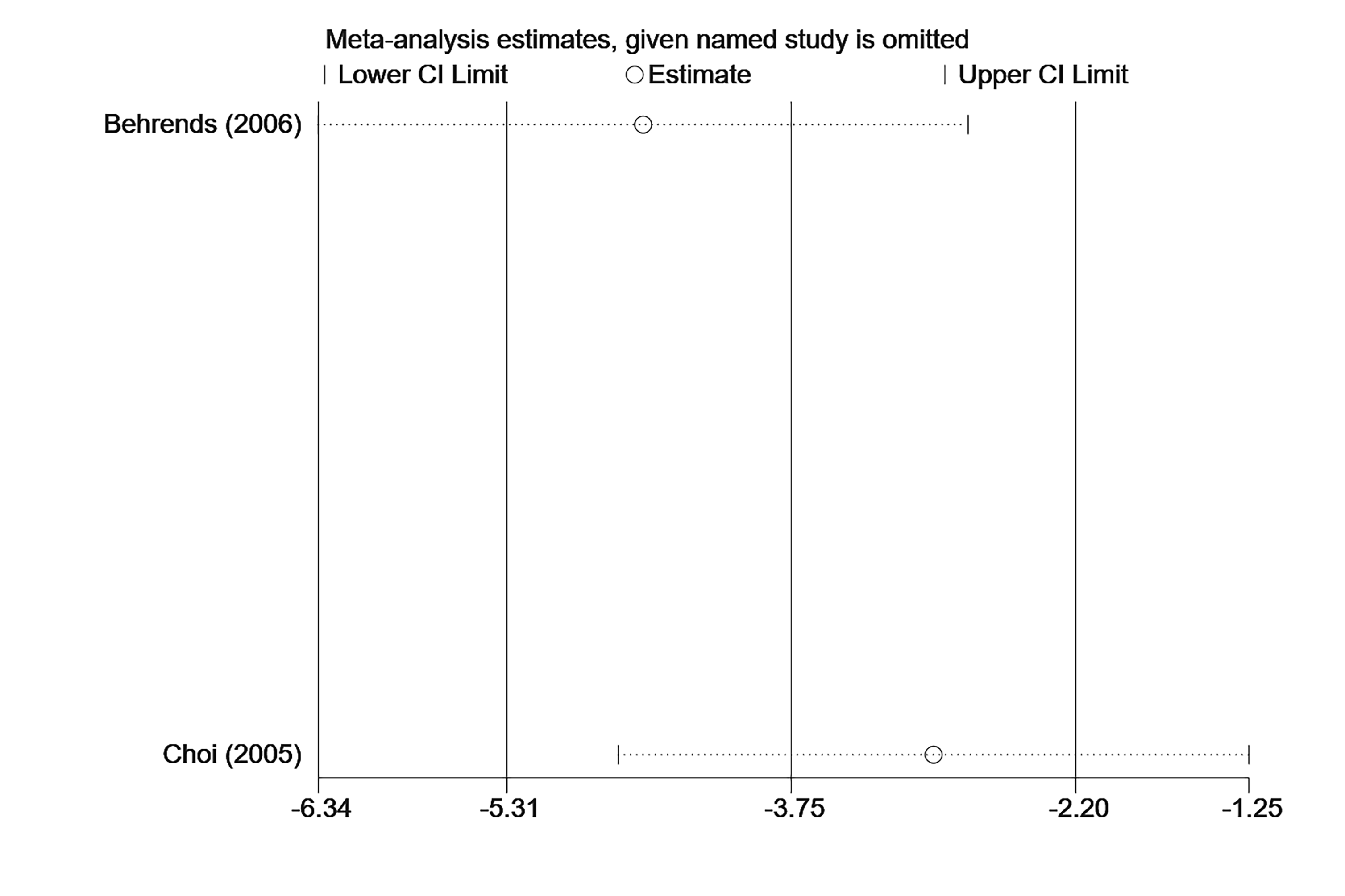
**

**S4. (A) Sensitivity analyses of ALT, (B) Sensitivity analyses of rat, (C) Sensitivity analyses of mice, (D) Sensitivity analyses of reperfusion time＞6h, (E) Sensitivity analyses of reperfusion time≤6h, (F) Sensitivity analyses of 32 degrees, (G) Sensitivity analyses of 34 degrees.**


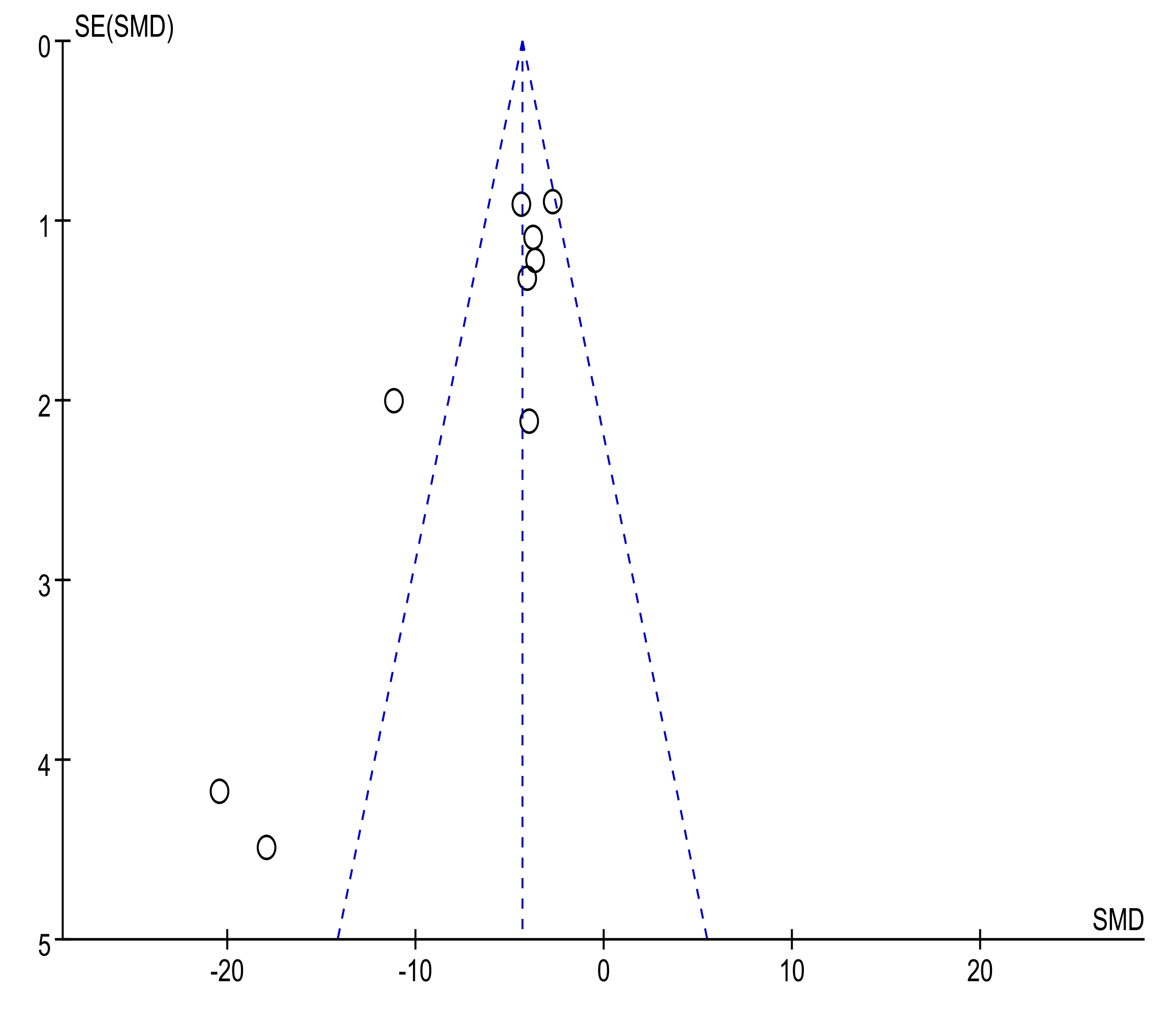
**S5. ALT's inverted funnel plot**
